# Prognostic factors related to all-cause mortality in very long-term follow-up of patients with heart failure: the REMADHE trial extended analysis

**DOI:** 10.1186/s12872-026-05671-6

**Published:** 2026-05-26

**Authors:** Edimar Alcides Bocchi, Guilherme Veiga Guimaraes, Cristhian Espinoza Romero, Silvia Moreira Ayub Ferreira, Bruno Biselli, Paulo Roberto Chizzola, Robinson Tadeu Munhoz, Julia Tizue Fukushima, André Rodrigues Durães, Leonardo Roever, Fátima das Dores Cruz

**Affiliations:** 1 Heart Failure Clinics, Instituto do Coração (Heart Institute), Faculdade de Medicina, Hospital das Clínicas HCFMUSP, Universidade de São Paulo, Rua Dr Melo Alves no 690, apto 41, São Paulo, CEP 01417-010 SP Brazil; 2 Unidade de Cardiologia, Hospital das Clínicas, Universidade Federal da Bahia, Salvador, Brazil; 3 Departament of Clinical Research – Brazilian Evidence, Uberlandia, Brazil

**Keywords:** Heart failure, Very long-term follow-up, Chagas disease, Prognosis, Renal

## Abstract

**Background:**

Disease management programs (DMP) have reduced hospitalizations and improved quality of life in heart failure (HF). However, prognostic factors and survival in very long-term follow-up (> 20 years) have not been reported.

**Aims:**

To evaluate the long-term effects of a disease management program (DMP) on heart failure outcomes and to identify prognostic predictors of all-cause mortality in patients with HF followed for up to 23.6 years.

**Methods:**

The REMADHE trial (NCT00505050, 2007-07-20) was a prospective, single-center, randomized trial (*n* = 412) comparing DMP versus usual care (C) with initial follow-up of 2.47 years. This extended analysis followed patients for 23.6 years to identify prognostic predictors of all-cause mortality.

**Results:**

The all-cause mortality rate was 88.3%. HF was the first cause of death followed by sudden death. Mortality was higher in the first 6-year follow-up. The predictive variables in multivariate analysis associated with mortality were age > 52 years (*P* = 0.015), Chagas etiology (*P* = 0.010), LVEF < 45% (*P* = 0.008), digoxin use (*P* = 0.002), NYHA IV (*P* = 0.01), blood urea nitrogen (BUN) (*P* = 0.03), and lymphopenia (*P* = 0.005). In very long-term follow-up, DMP did not affect mortality in patients under guideline-directed medical therapy (GDMT). HF as a cause of death was more frequent in the C group (41.0% vs. 33.3% in the DMP group; *P* < 0.02).

**Conclusions:**

DMP was not effective in reducing very long-term mortality; however, causes of death differed between groups, with more HF-related deaths in controls. Our findings that age, LVEF, Chagas’ disease, NYHA, renal function, lymphocytes, and digoxin use were associated with poor prognosis could influence future strategies to improve HF management.

**Graphical abstract:**

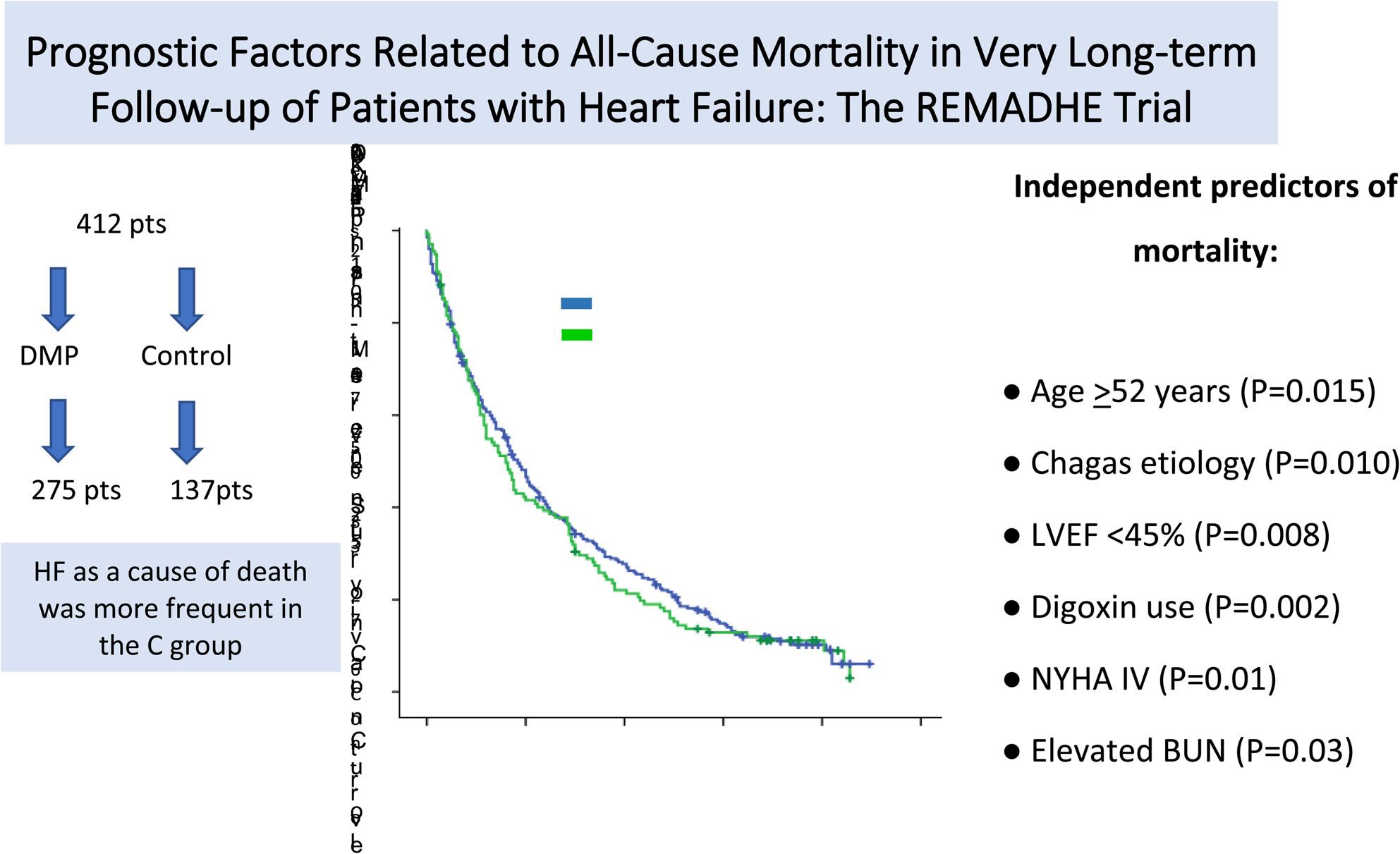

## Highlights

To our knowledge, this is the first study reporting prognostic factors in heart failure with follow-up exceeding 20 years

Independent mortality predictors included age >52 years, Chagas disease, LVEF <45%, digoxin use, NYHA IV, elevated BUN, and lymphopenia

Disease management programs (DMP) did not affect >20-year mortality when both groups received specialized heart failure clinic care

Distribution of causes of death differed between groups, with more heart failure deaths in the control group (usual care) and more sudden deaths in the DMP intervention group

## Introduction

Heart failure (HF) has an estimated prevalence of 1 to 4% of the global population [1] and remains associated with poor quality of life, high mortality, hospitalizations, and a substantial burden on the healthcare system. HF may have heterogeneous causes and pathways. However, HF trials and observational survival studies conducted worldwide have relatively limited short follow-up periods [2–4]. Few studies have assessed the long-term survival impact of HF beyond a 10-year period [5–11]. Thus, data regarding very long-term survival (> 20 years) and respective prognostic factors in HF are lacking. Heart failure (HF) has an estimated prevalence of 1 to 4% of the global population 1 and remains associated with poor quality of life, high mortality, hospitalizations, and a substantial burden on the healthcare system. HF may have heterogeneous causes and pathways. However, HF trials and observational survival studies conducted worldwide have relatively limited short follow-up periods [2–4]. Few studies have assessed the long-term survival impact of HF beyond a 10-year period [5–11]. Thus, data regarding very long-term survival (> 20 years) and respective prognostic factors in HF are lacking.

Educational and disease management programs (DMP) targeted at patients with HF have reported improvement in quality of life, and reduction in hospitalization and healthcare utilization [11–13]. However, doubt has been cast on the efficacy of these interventions in the medium and long term based on several published neutral studies [14]. In fact, very long-term efficacy of DMP in HF is unknown. Extended pivotal trials on treatment of HF have been shown heterogeneous concordance of the results in comparison with the first published trial [15–17]. Accordingly, also extended DMP trial development should be warranted.

The REMADHE trial was conducted initially during a mean follow-up period of 2.47 ± 1.75 years. The study demonstrated improvements in quality of life, reductions in hospitalizations and emergency visits among the DMP group, without statistical differences in mortality rates between the groups [13] The REMADHE trial had a smaller number of recruited patients in comparison with multicenter trials with consequent limited number of events mainly for mortality. A trial with a limited number of participants carries a considerable risk of failing to demonstrate a treatment difference when one is really present. Neutral trials may become positive with enhanced precision afford by the greater number of events over time. Accordingly, we studied the extended very long-term follow-up of the REMADHE trial to test if this DMP is effective in the scenario of more death events provided by the extended follow-up.

Understanding prognostic factors in patients with HF who survive very long periods is crucial for several reasons: (1) it provides realistic expectations for patient and family counseling; (2) it identifies modifiable risk factors that could be targeted for intervention; (3) it helps stratify risk for resource allocation in HF clinics; and (4) it generates hypotheses about mechanisms of long-term survival that can inform future therapeutic strategies. While both groups in our study received specialized HF care (an important design feature that allowed evaluation of prognostic factors under optimal management conditions), we hypothesized that DMP effects might persist through sustained improvements in self-care behaviors and patient empowerment over the extended follow-up period.

Therefore, the objective of our current study was to extend the follow-up period of patients initially included in the REMADHE trial up to 23.6 years. Also, we aimed to identify prognostic predictors of all-cause mortality in a population with HF who initially underwent education and telephone monitoring in a specialized and multidisciplinary HF unit.

## Methods

REMADHE was a prospective, single center, open trial with randomization 2:1, as previously detailed [13]. The REMADHE study compared the DMP group versus the Control group (C), in patients treated in a clinic specializing in HF with a multidisciplinary team. Patients in the DMP group underwent an educational program and continuous repetitive monitoring. Patients received reinforcement of education during the 2.47 ± 1.75-year follow-up at 6-month intervals. Education and monitoring were not repeated with frequent reinforcement throughout the very late follow-up. In this current extended study, we analyzed on June 2023 data of patients included in the period from October 1999 to January 18, 2005, with follow-up until 23.6 years.

Data about death were obtained from reports collected during medical visits, telephone calls, review of medical records, information from family members on data contained in the death certificate, research at the SEADE Foundation (State Data Analysis System), and the central deaths registry in Brazil (Ministry of Health). Death was classified as secondary to worsening HF or sudden death. Deaths that occurred in the hospital were classified as secondary to worsening HF based on review of available medical records, death certificates, and the clinical context of hospitalization. However, this approach: (1) has been used in other long-term HF registries; (2) is more reliable than classifying home deaths, which could represent either sudden death or unrecognized HF decompensation; and (3) represents the best available methodology for a 23.6-year retrospective follow-up [18].

### Ethics approval and consent to participate

The study protocol was submitted to the Heart Institute Ethical Committee receiving the number 827/99T. The local ethical committees approved the study that was performed in accordance with relevant guidelines/regulations. All patients or their legal guardians gave informed consent for participation in the study. The local ethical committees approved the extended long-term follow-up study. This trial was performed in accordance with the ethical principles outlined in the World Medical Association Declaration of Helsinki. The REMADHE study was registered at http://clinicaltrials.gov (Identifier NCT 00505050).

### Study population

The patients included in the very long-term follow-up of the REMADHE trial were initially recruited from a tertiary cardiology referral center who were undergoing outpatient follow-up with cardiologists specialized in heart failure (HF) at the Heart Failure Clinics. All patients were under guideline-directed medical therapy (GDMT). The eligibility criteria and exclusion criteria have been described previously [13].

### Statistical analysis

Descriptive statistics of quantitative variables were performed using mean (M), standard deviation (SD), and number of cases (N). Relative variations (Δ%) were also calculated and, if this was not possible, absolute variations (Δ) were evaluated between the results of the sequential follow-up of each variable. The distribution of quantitative variables was evaluated using the Kolmogorov-Smirnov test. Categorical variables were described with absolute and relative frequencies. Normality was determined by the Shapiro-Wilk test. The Student’s t-test was used to compare the baseline characteristics of groups C and DMP, and the Fisher exact test was used for unpaired values. In the analysis of mortality, the date of randomization up to the data obtained by telephone, by medical records or by death certificate was considered. Survival and event-free curves were calculated using the Kaplan-Meier method, and the log-rank test (Mantel-Cox) was used for comparison. *P* < 0.05 was considered statistically significant. The uncertainty measures of the statistical models were presented in the results, including 95% confidence intervals (CIs) for the hazard ratios (HRs), which directly reflect the uncertainties associated with the estimates. The procedures followed the software’s default methods, including well-established algorithms for proportional hazards analysis (Cox model) and stepwise variable selection in the multivariate model.

A univariate and multivariate proportional hazards model was adjusted to assess prognostic factors associated with mortality outcome. The following variables were tested initially on a univariate model: sex, age < or *≥* 52 years, ethnicity (white, black, mulatto), etiology (ischemic, hypertensive, alcoholic, chagasic, valvular, and others), diabetes type II, diabetes insulin-dependent, left ventricular ejection fraction (LVEF) *≥* or < 45%, left bundle-branch block, implanted pacemaker, digoxin use, New York Heart Association (NYHA), education level, marital status, quality of life (Minnesota Questionnaire), blood plasma levels of sodium, potassium, BUN, creatinine, glycemia, hemoglobin, leucocytes, thyroid hormones (T3/T4), thyroid stimulating hormone, and uric acid. Variables with *P* < 0.10 values were used to compose the multivariate model with a stepwise variable selection process. P values < 0.05 were considered significant. A baseline characteristic analysis was conducted to investigate potential confounding factors among the positive predictor variables examined in the multivariable analysis. Statistical analysis was performed with SPSS v 21 (SPSS Inc, Chicago, IL).

To address the potential overparameterization due to the limited number of events relative to the number of covariates, we employed the Stepwise selection method based on the Akaike Information Criterion (AIC). Additionally, we ensured that the event-to-variable ratio adhered, as much as possible, to the recommended threshold of at least 10 events per included variable to maintain the stability of the estimates. The categorization of continuous variables was based on both clinical and statistical criteria, taking into account the nature of the data and the context of the research. Continuous variables were categorized when clinically meaningful cut-offs were identified (e.g., widely accepted reference values in clinical practice) or when non-linear relationships between the variable and the outcome were observed. Furthermore, we performed checks for the proportional hazards assumption to ensure the validity of the fitted Cox model.

## Results

Groups DMP and C had similar demographic baseline characteristics, with a total of 412 included patients as previously published [15]. The time between the first randomization and outcome analysis was 23.6 years. The baseline characteristics of the patients were previously published in the initial study [13]. During the trial follow-up extended period the use of guideline-recommended medications and devices for the treatment of HF was strongly emphasized for all patients. In the last evaluation 70% of patients were receiving spironolactone, 84% beta-blocker, 67% renin-angiotensin-aldosterone system inhibitors, 26.8% angiotensin II receptor blockers, 1% angiotensin receptor-neprilysin inhibitor, 37% thiazides, 74% furosemide, 2.5% ivabradine, 35% hydralazine, and 32% nitrate. Also, in the last evaluation the percentage of patients with implanted pacemaker was 6.2% and for implanted cardioverter defibrillators were 3%. No statistical differences were observed between the groups.

**Fig. 1:**
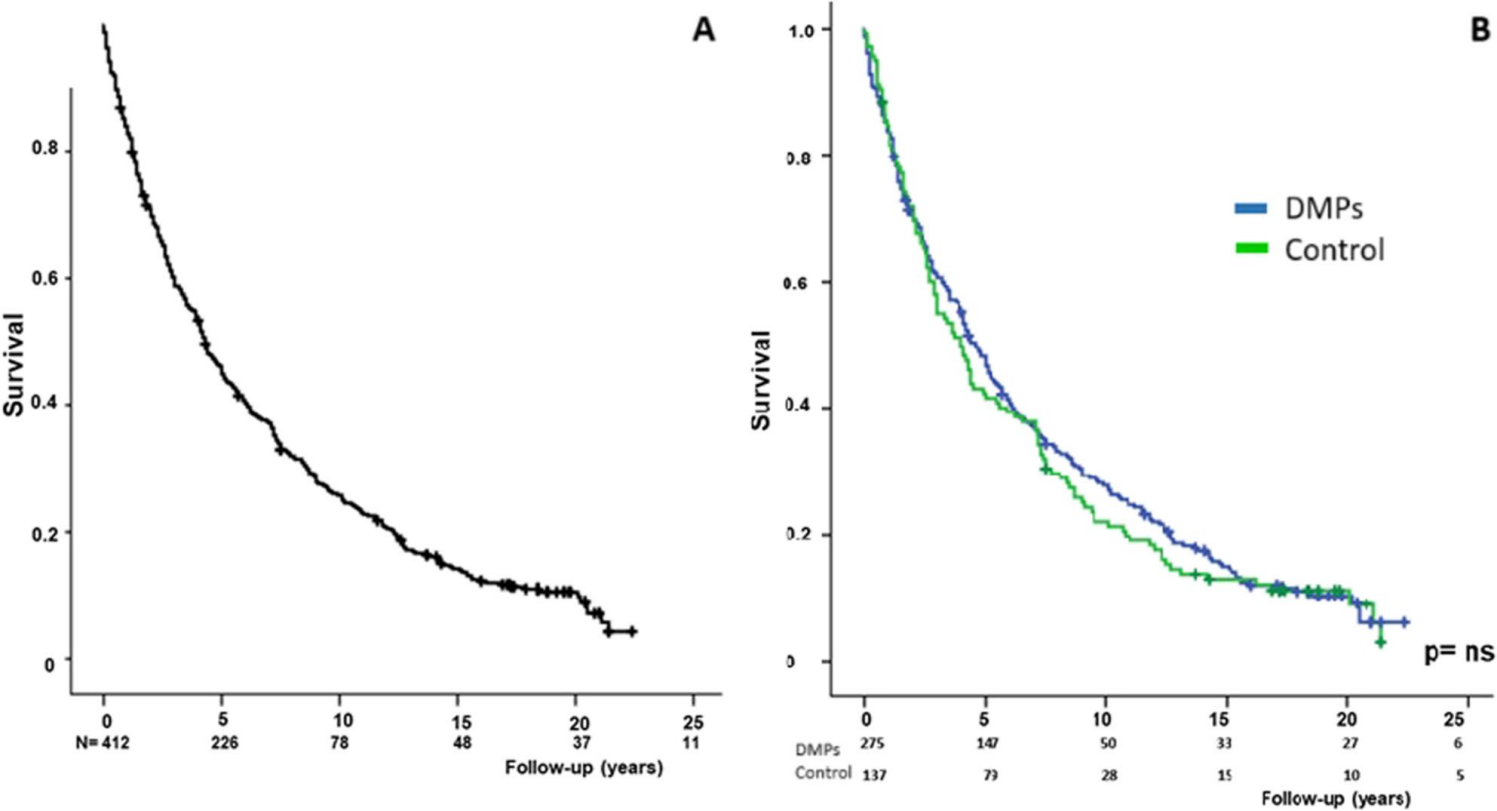
Kaplan-Meier Survival Curve in the Total Population **A**. The Survival Curves According to Intervention and Usual Care Groups **B**

**Fig. 2:**
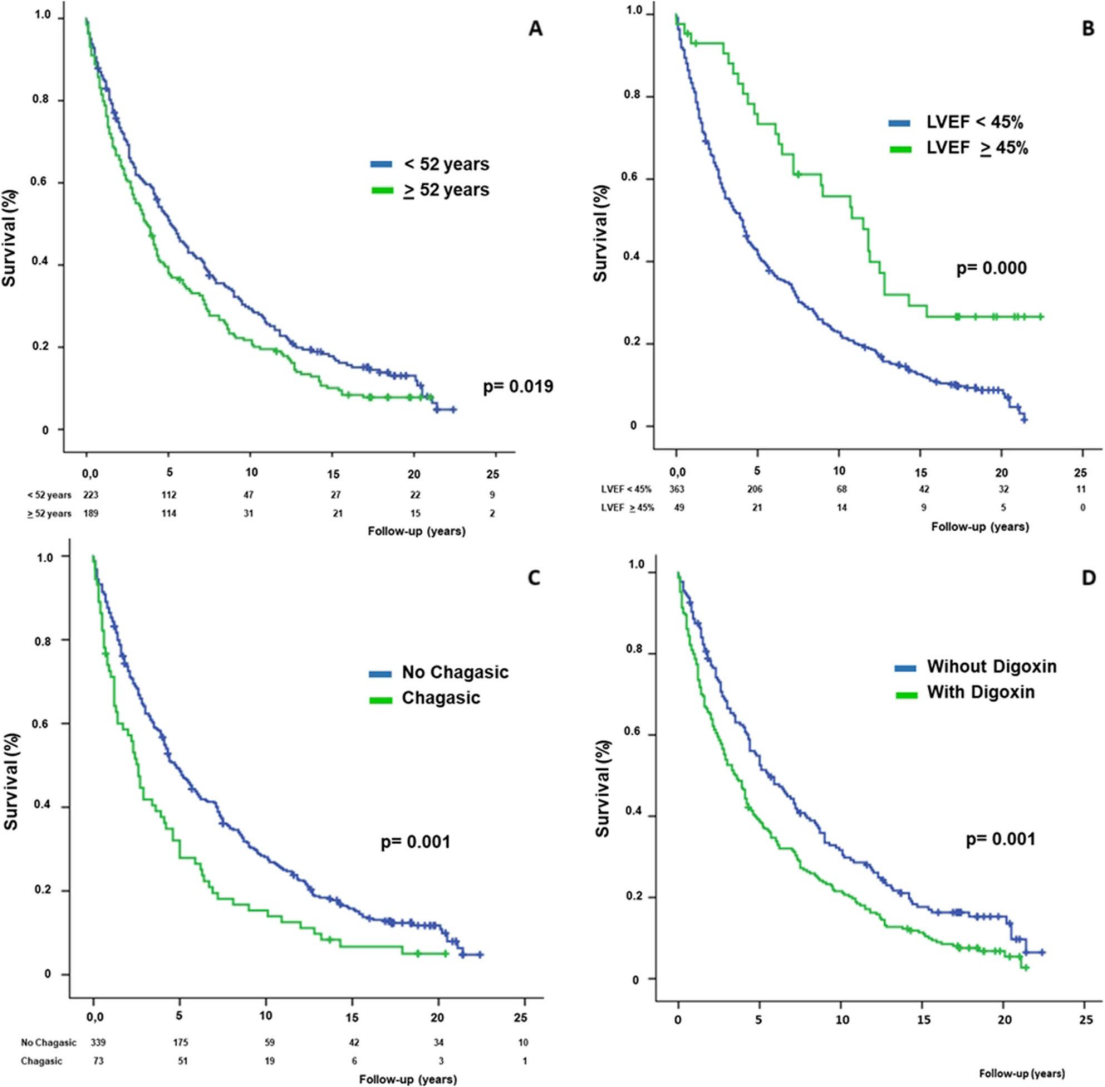
Kaplan-Meier Survival Curve in Subgroup Analysis; **A** According to Age < 52 and *≥* 52 Years; (**B**) According to Left Ventricular Ejection Fraction < 45% and *≥* 45%; (**C**) According to Chagas’ and non-Chagas’ Etiology; (**D**) According to Digoxin Use

**Fig. 3:**
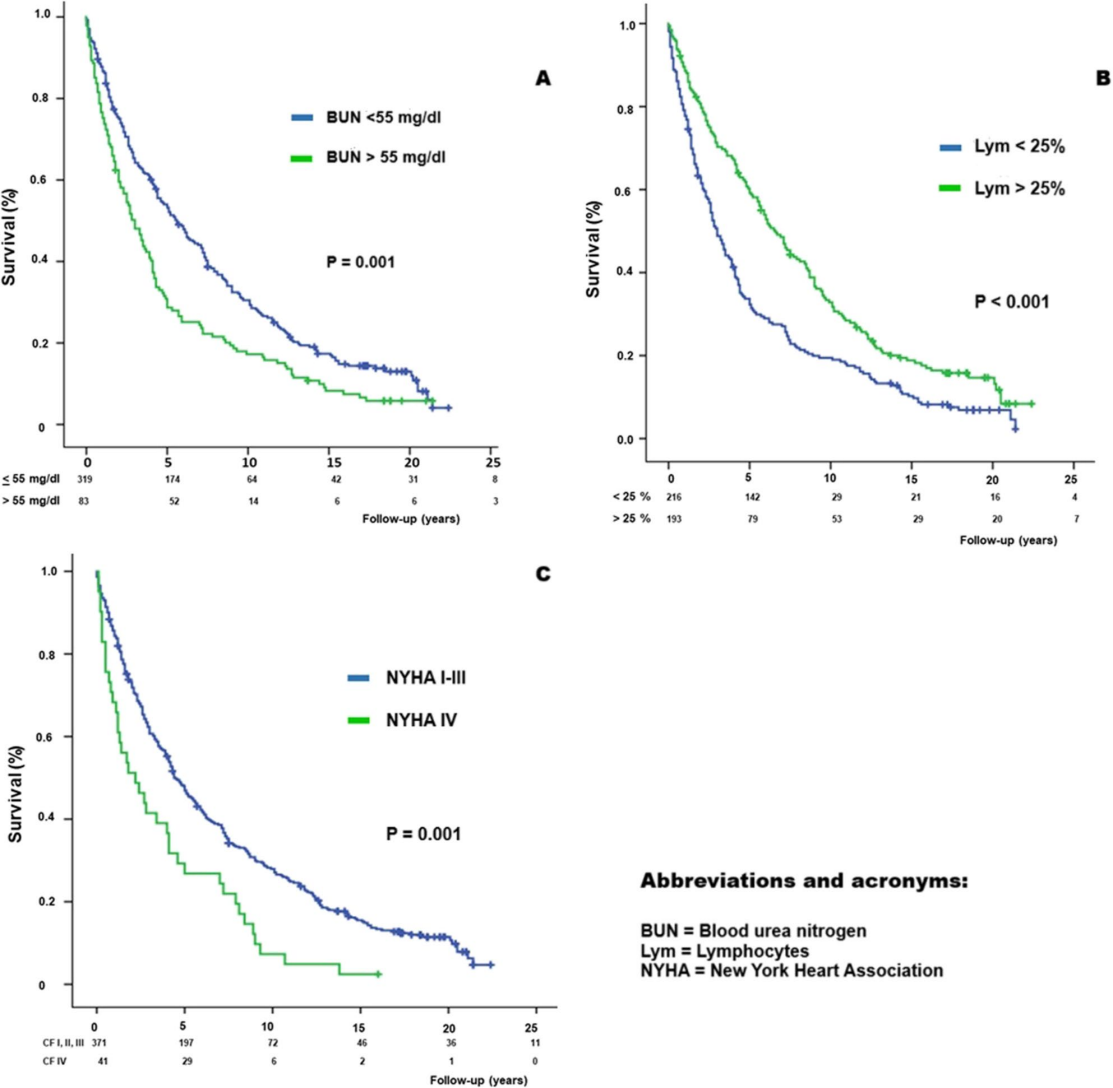
Kaplan-Meier Survival According to Subgroup Analysis: (**A**), According to BUN *≤* 55 mg/dl and > 55 mg/dl; (**B**), According percentage of Lymphocytes > 25% versus *≤* 25%; (**C**), According New York Association NYHA

**Fig. 4:**
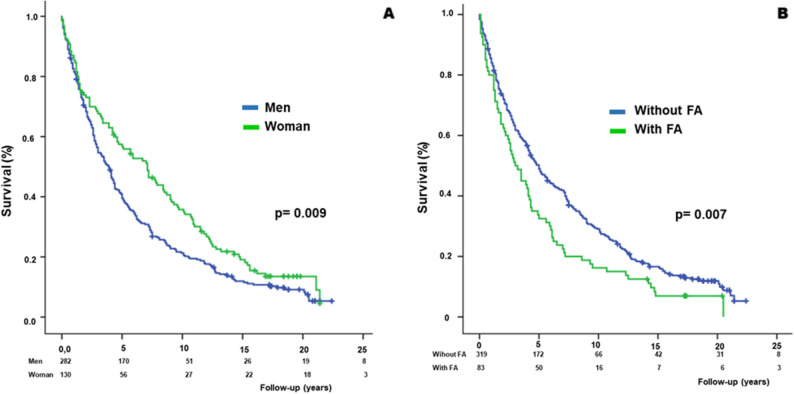
Kaplan-Meier Survival Curve: (**A**) According Sex Men and Woman, (**B**) and Kaplan-Meier Survival Curve According Patients With Atrial Fibrillation

Mortality data were analyzed from October 1999 to June 2023, showing all-cause mortality rate of 88.3% (Fig. 1A). HF was the cause of death in 35.9% (*n* = 132) of patients who died; 25.5% (*n* = 105) died at home, other causes of death were observed in 19.3% (*n* = 79), and in 11.2% (*n* = 46) the cause was unknown. The survival curve showed a steeper decline during the first 6 years of follow-up compared to subsequent years (Fig. 1A). The survival curves according to DMP and C groups are shown in Fig. 1B. At 23.6-year follow-up, univariate analysis revealed that several variables were associated with lower survival rates (Table 1), including age *≥* 52 years (Fig. 2A), LVEF < 45% (Fig. 2B), chagasic etiology (Fig. 2C), digoxin users (Fig. 2D), BUN (Fig. 3A), lymphocytes (Fig. 3B), and NYHA IV (Fig. 3C), male sex (Fig. 4A), and atrial fibrillation (AF) (Fig. 4B). On the multivariate analysis, the predictive variables for mortality were age *≥* 52 years (HR 1.315; 95% Confidence Interval [CI], 1.055 to 1.640; *P* = 0.015); Chagas etiology (HR 1.672; 95% CI, 1.252 to 2.232; *P* < 0.001); LVEF > 45% as reference (HR 0.582; 95% CI, 0.389 to 0.870 for LVEF < 45%; *P* = 0.008), indicating higher mortality risk with reduced LVEF; use of digoxin (HR 1.425; 95% CI, 1.138 to 1.785; *P* = 0.002); NYHA IV (HR 1.604; 95% CI, 1.122 to 2.295; *P* = 0.010); elevation of BUN (HR 1.008; 95% CI, 1.003 to 1.014; *P* = 0.038); and lymphocytes > 25% as reference (HR 0.772; 95% CI, 0.641 to 0.929 for lymphopenia; *P* = 0.005), indicating higher mortality with lymphopenia.

**Table 1:** Univariable Analysis of Predictors Associated With Any Mortality at 23.7-year follow-up

Variable	Total	Death, *n* (%)		HR (95% CI)	*P*
		No	Yes		
	*N* = 412 (%)	81 (20)	331 (80)		
Group					
DMPs	276 (67)	44 (68.8)	232 (66.7)		
C	136(33.0)	20 (31.3)	116 (33.3)	1.074 (0.859 to 1.343)	0.529
Transplantation	30 (7.3)	8 (12.5)	22 (6.3)	0.681 (0.442 to 1.050)	0.082
Sex (men)	**282 (68.4)**	**36 (56.3)**	**246 (70.7)**	**1.395 (1.106 to 1.758)**	**0.005**
Age (*≥* 52y)	**189 (45.9)**	**22 (34.4)**	**167 (48)**	**1.928 (1.051 to 1.603)**	**0**,**015**
Race					0,756
White	225 (54.6)	38 (59.4)	187 (53.7)		
Mulatto	105 (25.5)	14 (21.9)	91 (26.1)		
Black	82 (19.9)	12 (18.8)	70 (20.1)		
Race (Nonwhite)	186 (45.1)	37 (45.7)	149 (45)	1.019 (0.825 to 1.259)	0.859
Etiology					**0**,**011**
Ischemic	116 (28.2)	17 (26.6)	99 (28.5)		
Hypertensive	65 (15.8)	14 (21.9)	51 (14.7)	0.723 (0.516 to 1.015)	0.061
Alcoholic	18 (4.4)	2 (3.1)	16 (4.6)	1.540 (0.907 to 2.614)	0.110
Idiopathic	100 (24.3)	14 (21.9)	86 (24.8)	1.088 (0.814 to 1.453)	0.570
Chagasic	**73 (17.8)**	**8 (12.5)**	**65 (18.7)**	**1.469 (1.073 to 2.010)**	**0.016**
Valvular	13 (3.2)	2 (3.1)	11 (3.2)	0.826 (0.443–1.541)	0.549
Congenital	3 (0.7)	1 (1.6)	2 (0.6)	0.804 (0.198 to 3.260)	0.760
Postpartum	4 (1.0)	2 (3.1)	2 (0.6)	0.362 (0.089 to 1.467)	0.155
Others	15 (3.7)	3 (3.1)	12 (3.2)	0.861 (0.462 to 1.607)	0.639
Hypertrophic	3 (0.7)	2 (3.1)	1 (0.3)	0.250 (0.035 to 1.794)	0.168
DM non insulin dependent	85 (20.6)	13 (20.3)	72 (20.7)	0.930 (0.717 to 1.205)	0.582
DM insulin dependent	19 (4.6)	4 (6.3)	15 (4.3)	0.786 (0.467 to 1.322)	0.364
LVEF (*≥* 45%)	**43 (10.6)**	**15 (24.2)**	**28 (8.2)**	**0.485 (0.329 to 0.714)**	**< 0.001**
LBBB	80 (19.9)	9 (14.3)	71 (20.9)	1.151 (0.886 to 1.496)	0.291
AF	**81 (20.1)**	**9 (14.3)**	**72 (21.2)**	**1.365 (1.052 to 1.773)**	**0.019**
PM	20 (5.0)	2 (3.2)	18 (5.3)	1.444 (0.897 to 2.323)	0.130
Digoxin use	**23 (56.4)**	**24 (38.1)**	**206 (59.7)**	**1.44 (1.161 to 1.787)**	**0.001**
NYHA NYHA, n (%)					**0.002**
I	61 (14.8)	16 (19.8)	45 (13.6)		
II	200 (48.5)	41 (50.6)	159 (48)	1.200 (0.868 to 1.658)	0.270
III	110 (26.7)	21 (25.9)	89 (26.9)	1.288 (0.906 to 1.830)	0.158
IV	**41 (10)**	**3 (3.7**	**38 (11.5)**	**2.196 (1.438 to 3.353)**	**< 0.001**
Sodium mmol/l, n (range)	139 (137–141)	139 (137–140)	139 (136–141)	0.997 (0.987 to 1.008)	0.577
Potassium, mmol/l	4.5 (4.2–4.9)	4.5 (4.2–4.78)	4.5 (4.1–4.9)	0.998 (0.956 to 1.042)	0.934
BUN, mg/dl	**47 (36–63)**	**39 (32–54)**	**48 (37–65)**	**1.010 (1.005 to 1.016)**	**< 0.001**
BUN > 55 mg/dl (32%)		5 (23.8)	80 (34.2)	1.308 (0.997 to 1.715)	0.052
Creatinine mg/dl	**1.1 (1-1.4)**	**1.0 (0.9–1.3)**	**1.2 (1-1.4)**	**1.044 (1.007 to 1.081)**	**0.019**
Glucose, mg/dl	100 (91–116)	102 (94–113)	100 (91–116)	0.998 (0.996 to 1.001)	0.160
Hemoglobin g/dl	14 (13–15)	14 (13–15)	14 (13–15)	0.960 (0.910 to 1.013)	0.137
Hematocrit, %	42 (38–45)	42 (38–46)	42 (38–45)	0.991 (0.973 to 1.009)	0.304
Leukocytes x10^3^	7.30 (6.10–8.80)	7.05 (5.83–8.18)	7.40 (6.20–8.90)	1.033 (0.989 to 1.079)	0.141
Lymphocytes x10^3^	**1.76 (1.33–2.26)**	**2.04 (1.55–2.45)**	**1.72 (1.30–2.24)**	**0.677 (0.575 to 0.796)**	**< 0.001**
Lymphocytes > 25% (50.8%)		**36 (75.0)**	**172 (47.6)**	**0.611 (0.496 to 0.753)**	**< 0.001**
T4, ng/dl	1.4 (1.2–1.5)	1.3 (1.1–1.6)	1.4 (1.2–1.5)	0.953 (0.586 to 1.550)	0.846
TSH, µmol/L	2.0 (1.2–3.3)	1.7 (1.1–2.5)	2.1 (1.2–3.4)	1.012 (0.996 to 1.028)	0.145
Uric Acid, mg/dl	7.9 (6.03–9.48)	6.35 (4.7–8.1)	8.2 (6.4–9.63)	1.001 (0.998 to 1.003)	0.632

*DMPs* Disease management program, *C* Usual care, *DM* Diabetes mellitus type 2, *LVEF* Left ventricular ejection fraction, *LBBB* Left bundle-branch block, *AF* Atrial fibrillation, *PM* pacemaker, *NYHA* New York Heart Association; Data with statistical significance are in bold

Chagas’ disease did not show a statistically significant difference in causes of death compared with other etiologies (*P* = 0.07). In death from Chagas’ disease, HF was the cause in 43.2%, sudden death was observed in 17.6%, other causes in 33.8%, and unknown in 5.4%. In non-Chagas’ disease deaths, HF was the cause in 37%, sudden death was observed in 34.6, other causes in 17.1, and unknown in 11.3%. Causes of death were different according to baseline LVEF < 45% and *≥* 45% (*P* < 0.04). In LVEF < 45% HF as a cause of death, sudden death, other causes, and unknown causes were 40, 29.7%, 21.7%, and 8.6%, respectively. In LVEF *≥* 45%, HF cause of death, sudden death, other causes, and unknown causes were 16.7%, 29.7%, 21.7%, and unknown in 8.6% respectively. Causes of death were different according to baseline BUN > 55 mg/dl and BUN *≤* 55 mg/dl (*P* < 0.01). In BUN > 55 mg/dl, HF as the cause of death, sudden death, other causes, and unknown causes were 46%, 24.2%, 18.5%, and 11.3%, respectively. In BUN *≤* 55 mg/dl, HF as the cause of death, sudden death, other causes, and unknown causes were 34%, 35%, 21.8%, and 9.2%, respectively. Other independent variables related to mortality in multivariate analysis did not influence the causes of death.

The mean survival was 6.2 ± 0.52 years in C versus 6.6 ± 0.37 years in DMP (*P* = 0.656) up to 23.6-year follow-up (Fig. 1B). HF as a cause of death and sudden death were different between groups DMP and C (*P* < 0.02). HF during hospitalization was the cause of death in 33.3% and 41% in DMP and C groups, respectively; and sudden death was observed in 28.4% and 20.4% of deaths in DMP and C groups, respectively. Other causes of death or unknown causes were observed in 34.7%, and 34.2% of the deaths in DMP and C groups, respectively (P = ns).

## Discussion

One of the notable strengths of this study is the very long-term follow-up of patients with HF, which, to the best of our knowledge, represents the first DMP analysis of a follow-up period exceeding 20 years. The main findings can be summarized as follows: (1) the survival of HF patients analyzed over a 20-year period showed during the first 6 years an inclination of the survival curve suggesting initial high risk even in patients under ambulatory care; (2) age (*≥* 52 years), Chagas disease, LVEF < 45%, digoxin use, NYHA IV, elevated BUN, and lymphopenia were independent predictors of mortality; (3) DMP and C groups had similar survival. However, HF as cause of death was more frequent in C; (4) HF was the first cause of death followed by sudden death; (5) Some independent variables on multivariate analysis were associated with different modes of death, including Chagas’ disease; baseline LVEF and renal function. While extended follow-up provided valuable insights, interpretations were tempered to reflect limitations of categorization, stepwise selection, potential misclassification of modes of death, and exposure dilution.

To our knowledge, this study is novel in the analysis of very long-term mortality (exceeding 20 years) in HF patients who underwent DMP. Our results showed better HF survival in comparison with recently reported HF data up to 10-year follow-up [5]. One reason for this would be that our patients were followed up by HF specialists in a Heart Failure Clinic. Also, our findings add new data on modes of death in very long-term follow-up on HF. Mechanisms related to higher mortality for approximately the first 6 years are unknown. The higher mortality up to 5 years was also reported recently after HF hospitalization. Those who responded poorly or not at all to triple therapy including those who did not maintain the initial response could have died in the first years instead of those who responded to therapy and had longer follow-up. Patient characteristics under optimized therapy as observed in our results could influence the response to treatment. As main implications of our results independent modifiable markers with a strong pathophysiological rationale could be priority targets for treating or planning research on HF in very long-term follow-up. Renal function and LVEF seem to have these characteristics.

We found in the literature only one publication that reported the etiology, prevalence and mortality from HF in the European part of Russia over a very long period of 20 years. The authors found that the median survival time was 8.4 years in patients with NYHA I–II and 3.8 years in patients with NYHA III–IV [19].

One important finding requiring careful interpretation is that REMADHE DMP did not affect very long-term mortality. Several factors likely explain this result: (1) Both DMP and control groups received continuous specialized care in a multidisciplinary Heart Failure Clinic with GDMT optimization throughout the 23.6-year follow-up—this is a unique and important design feature of our study; (2) The intensive structured education and monitoring reinforcement that characterized the DMP intervention was maintained primarily during the initial trial period (2.47 years) and not systematically continued with the same frequency and structure during the extended follow-up, leading to dilution of the intervention effect over time; (3) The finding that both groups had similar survival likely reflects the high-quality specialized care both groups received rather than inefficacy of DMP per se. However, the observation that the control group had more HF-related deaths while the DMP group had more sudden deaths suggests the initial DMP intervention may have had some lasting effect on disease trajectory, potentially by improving HF progression outcomes but not preventing sudden death. This differential effect on modes of death has important clinical implications for long-term HF management strategies.

The prognostic factors we identified (age, Chagas disease, LVEF, renal function, lymphocytes, digoxin use) are particularly valuable because they were predictive even in a cohort receiving optimal specialized care. This suggests these factors represent fundamental disease severity markers or non-modifiable characteristics that remain important regardless of quality of care. Identifying these factors has important implications for long-term risk stratification, resource allocation, and patient counseling.

Unfortunately, systematic quality of life and hospitalization data were not collected during the extended follow-up period beyond the original 2.47-year trial. This represents an important limitation of our study and an area that warrants investigation in future prospective very long-term HF studies. Understanding whether the early benefits of DMP on quality of life and hospitalizations persist, attenuate, or resolve over very long-term follow-up would provide valuable information for designing sustained intervention programs.

Worse prognosis of chagasic HF on shorter follow-up was also observed in the extended long-term follow-up [13]. Despite already being first described in 1994 by Bocchi et al., the mechanisms related to worse prognosis in chagasic HF still is an unresolved issue [20]. The complex pathogenesis and physiopathology comprising persistent myocarditis with fibrosis, parasite persistence with inflammatory response, autoimmunity, damage to the parasympathetic system causing sympathetic over activity, microvascular abnormalities, conduction system abnormalities, brady- and tachyarrhythmias, biventricular dilated cardiomyopathy, apical aneurysm, thromboembolism, or remodeled ventricles may be related to worse prognosis [21, 22]. Also, only 35.8% of patients with Chagas disease were receiving baseline beta-blocker therapy. However, the lack of knowledge about whether GDMT is effective for chagasic HF may have influenced the smaller proportion of beta-blocker therapy compared with other etiologies. Medical treatment has been extrapolated from trials that included other etiologies or studies with limited design [22]. However, a subanalysis of the REMADHE trial showed that the survival of patients with Chagas disease undergoing beta-blocker therapy was similar to that of other etiologies [23, 24].

Our results on multivariate analysis concerning age align with findings of studies that reported a negative impact of aging on survival [5]. However, in our cohort, patients were relatively younger (mean age of 51 years), and an age already *≥* 52 years was associated with lower survival. The presence of a younger population can be attributed to earlier manifestation of etiologies such as Chagas’ disease, valvar abnormalities, and limited access to prevention in a population despite risk factors of developed and undeveloped countries [25].

Our findings on very long-term follow-up are in agreement with prior studies showing that reduced LVEF is a well-established predictor of HF mortality particularly with an average follow-up of up to 5 years [26–28]. Studies have not explored very longer follow-up periods. Otherwise, heart failure with LVEF > 45% (HFpEF) was also associated with increased mortality mainly in NYHA IV [29, 30]. However, prognosis of HFpEF is controversial depending of characteristics of included patient in studies. Overall, it is expected patients with recovered LVEF in HFpEF group. Better prognosis was reported in HF with improved LVEF in comparison with persistent HFpEF, declined EF and persistent heart failure with reduced ejection fraction [26]. Additionally, the worsening of functional class is known to be associated with worse outcomes in HF, which was consistent with our findings in very long-term follow-up. NYHA IV was also associated with reduced survival, similar to observations from other studies with follow-up periods of up to 10 years [29, 30].

Concerning the digoxin association with worse prognosis reported in our results, it is crucial to highlight that studies had reported contradictory associations of digoxin with mortality in HF [31–33]. However, most studies have the caveat of absence of serum digoxin levels assessment, which might have affected outcomes. Subanalysis of the Digitalis Investigation Group trial showed a linear dose–response relationship linking serum concentration to mortality [34]. Also, the reason for digoxin prescription may be a confounder because in clinical practice digoxin could had been prescribed for more seriously ill patients. These findings suggest that digoxin use warrants careful consideration in HF patients with very long-term follow-up, given that an association with increased mortality has been reported in previous research and was observed in our results; however, confounding by indication cannot be excluded given the observational nature of this analysis. Also, the evidence of benefits of digoxin may be limited in patients undergoing contemporaneous HF treatment.

Our data in which the baseline mean urea values > 55 mg/dl were associated with reduced survival confirms previous publications, however, adding new very long-term data. Numerous studies, particularly in the context of decompensated HF, have examined the prognostic value of elevated BUN (> 55–80 mg/dl) as a predictor of morbidity and mortality, albeit with short-term follow-up [35, 36]. Report of the Swedish Heart Failure Kidney Registry showed that renal dysfunction is common and strongly associated with short-term and long-term outcomes up to 10-year follow-up in patients with HF [36]. Systematic review and meta-analysis reported that worsening renal function predicts substantially higher rates of mortality and hospitalization in patients with HF [37].

Baseline lymphopenia as a biomarker for prognosis in HF has been reported, but it has not yet been demonstrated in long-term follow-up as in our results [38–40]. Lymphopenia is also a marker for worse prognosis in other systemic diseases including COVID-19 [41]. The mechanisms responsible for the increment in the relative reduction in lymphocytes in HF are not fully understood. An increase in neutrophil because of systemic inflammation, and lymphopenia caused by elevated cytokines, splanchnic congestion, apoptosis, increased endogenous cortisol and sympathetic tone may play a role [42]. HF can trigger a significant increase in systemic cortisol production [43].

The findings from this very long-term follow-up study have several important clinical implications: First, risk stratification - the identified prognostic factors (age > 52 years, Chagas disease, LVEF < 45%, elevated BUN, lymphopenia, NYHA IV, and digoxin use) can be used to develop comprehensive long-term risk stratification tools for HF patients. These factors remained predictive even in patients receiving optimal specialized care, suggesting they represent fundamental markers of disease severity; Second, modifiable risk factors - among the identified predictors, renal function represents a potentially modifiable risk factor that should be aggressively monitored and managed in HF patients. Strategies to preserve renal function, avoid nephrotoxic agents, and optimize GDMT despite mild renal impairment may improve very long-term outcomes; Third, the Chagas cardiomyopathy - patients with Chagas disease require special attention given their consistently worse prognosis. Fourth, the relevance of specialized HF clinic care - the relatively good survival observed in both groups underscores the importance of long-term specialized multidisciplinary HF clinic care. Even without intensive DMP, continued specialized care with GDMT optimization appears crucial for long-term outcomes; Finally, these data provide realistic survival expectations for counseling HF patients and their families. Some HF patients, particularly those under specialized care with favorable prognostic profiles, can survive > 20 years, which is important information for shared decision-making regarding advanced therapies, palliative care discussions, and life planning.

Our study has several important limitations that should be considered when interpreting the results: First, being conducted at a single specialized tertiary HF center limits generalizability to other settings, particularly community-based practices or centers without specialized multidisciplinary HF programs. Second, this extended follow-up was not pre-specified in the original trial design, which limits causal inferences and introduces potential biases inherent to retrospective analyses. Third, the very long-term nature of follow-up necessitated pragmatic approaches to death classification. Hospital deaths were classified as HF-related, which may have resulted in some misclassification. Cause of death determination became increasingly challenging over the 23.6-year period. Fourth, quality of life, hospitalization rates, medication adherence, and other clinical parameters were not systematically collected during the extended follow-up period beyond the initial 2.47 years. This limits our ability to understand trajectory of these important outcomes. Fifth, despite comprehensive baseline assessment, unmeasured confounders accumulated over 23.6 years (e.g., changes in social support, socioeconomic status, access to care, development of comorbidities) may have influenced outcomes. Finally, by definition, very long-term follow-up studies the characteristics of survivors. The cohort alive at later time points represents a selected population that may not be representative of all HF patients and temporal changes in HF management (survival bias).

## Conclusion

In conclusion, DMP was not effective in reducing very long-term mortality; however, causes of death differed between groups, with more HF-related deaths in controls. Our findings that age, LVEF, Chagas’ disease, NYHA, renal function, lymphocytes, and digoxin use were associated with poor prognosis and could influence future strategies to improve HF management. This study would be highly valuable for patients, doctors, and healthcare professionals seeking a comprehensive understanding and strategies for management of HF in very long-term follow-up.

## Data Availability

Data are available upon reasonable request. If someone wants to request the data from this study, the contact will be Prof Dr Edimar Bocchi [dcledimar@incor.usp.br](mailto: dcledimar@incor.usp.br).

## Abbreviations

HF: Heart Failure
DMP: Educational and disease management programs
REMADHE trial: Long-Term Prospective, Randomized, Controlled Study Using Repetitive Education at Six-Month Intervals and Monitoring for Adherence in Heart Failure Outpatients
C: Control group
LVEF: Left ventricular ejection fraction
GDMT: Guideline-directed medical therapy
BUN: Blood urea nitrogen
NYHA: New York Heart Association
CI: Confidence interval
HR: Hazard ratio
AF: Atrial fibrillation
